# Decision Aids for Patients With Head and Neck Cancer: Qualitative Elicitation of Design Recommendations From Patient End Users

**DOI:** 10.2196/43551

**Published:** 2023-06-05

**Authors:** Eleah Stringer, Julian J Lum, Jonathan Livergant, Andre W Kushniruk

**Affiliations:** 1 School of Health Information Science University of Victoria Victoria, BC Canada; 2 Nursing and Allied Health Research and Knowledge Translation BC Cancer Vancouver, BC Canada; 3 Trevor and Joyce Deeley Research Centre BC Cancer - Victoria Victoria, BC Canada; 4 Department of Biochemistry and Microbiology University of Victoria Victoria, BC Canada; 5 Radiation Oncology BC Cancer - Victoria Victoria, BC Canada; 6 Faculty of Medicine University of British Columbia Vancouver, BC Canada

**Keywords:** decision support, decision aid, app design, oncology, head and neck cancer, patient information needs, qualitative

## Abstract

**Background:**

Patients with head and neck cancer (HNC) carry a clinically significant symptom burden, have alterations in function (eg, impaired ability to chew, swallow, and talk), and decrease in quality of life. Furthermore, treatment impacts social activities and interactions as patients report reduced sexuality and shoulder the highest rates of depression across cancer types. Patients suffer undue anxiety because they find the treatment incomprehensible, which is partially a function of limited, understandable information. Patients’ perceptions of having obtained adequate information prior to and during treatment are predictive of positive outcomes. Providing patient-centered decision support and utilizing visual images may increase understanding of treatment options and associated risks to improve satisfaction with their decision and consultation, while reducing decisional conflict.

**Objective:**

This study aims to gather requirements from survivors of HNC on the utility of key visual components to be used in the design of an electronic decision aid (eDA) to assist with decision-making on treatment options.

**Methods:**

Informed by a scoping review on eDAs for patients with HNC, screens and visualizations for an eDA were created and then presented to 12 survivors of HNC for feedback on their utility, features, and further requirements. The semistructured interviews were video-recorded and thematically analyzed to inform co-design recommendations.

**Results:**

A total of 9 themes were organized into 2 categories. The first category, eDAs and decision support, included 3 themes: familiarity with DAs, support of concept, and versatility of the prototype. The second category, evaluation of mock-up, contained 6 themes: reaction to the screens and visualizations, favorite features, complexity, preference for customizability, presentation device, and suggestions for improvement.

**Conclusions:**

All participants felt an eDA, used in the presence of their oncologist, would support a more thorough and transparent explanation of treatment or augment the quality of education received. Participants liked the simple design of the mock-ups they were shown but, ultimately, desired customizability to adapt the eDA to their individual information needs. This research highlights the value of user-centered design, rooted in acceptability and utility, in medical health informatics, recognizing cancer survivors as the ultimate knowledge holders. This research highlights the value of incorporating visuals into technology-based innovations to engage all patients in treatment decisions.

## Introduction

Head and neck cancer (HNC) is the sixth leading type of cancer by incidence worldwide and diagnosed in approximately 4300 Canadians per year [1]. Based on clinical and pathological manifestation, patients with HNC may be provided an option of surgery, radiation, chemotherapy, immunotherapy, and combinations thereof, each influencing morbidity differently [2]. For patients to participate in their care, they must comprehend their disease and treatment options, consider their own preferences, participate in decision-making to the degree they wish, and make a decision consistent with personal preferences. Patients who direct decisions, even if more than anticipated, fare better on all decision-related outcomes, emphasizing the need for oncologists and surgeons to endorse, facilitate, and support patient participation in treatment decision-making [3].

Some patients with HNC may struggle to take part in decision-making regarding treatment options because of the complexity of the information that needs to be accurately conveyed and understood [4,5]. This can be particularly challenging in the context of a globalized world consisting of cultural differences and varying health literacy levels, increasing the need for enhanced transparent communication of risk and outcomes associated with treatments [5,6]. Furthermore, research on risk literacy in medical decision-making shows that across different cultures, people often struggle to grasp the prerequisite concepts necessary for understanding health-related risk information such as numbers, graphs, and basic medical facts. Errors occur because inappropriate information formats complicate and mislead adaptive decision makers [6].

Decision aids (DAs) “are interventions that support patients by making their decisions explicit, providing information about options and associated benefits/harms, and helping to clarify congruence between decisions and personal values” [7]. DAs complement, rather than replace, counseling from a health care practitioner as an interactional strategy to facilitate patient involvement and contribute to patient concordance [4]. DAs are useful when the best treatment strategy depends on a preference for the benefit-harm trade-off inherent in a particular choice.

A scoping review conducted on electronic DAs (eDAs) for patients with HNC returned 12 relevant articles that discussed 5 different patient eDAs [8]. The scoping review confirmed the value of eDAs in this population supporting “further research and development in this area.” The scoping review did not, however, reveal detailed technical features of existing eDAs. The patient eDA developed by Petersen et al [9] remained available online, so features were viewable, but the other 4 eDAs were not available for viewing. The aim of this study is to respond to the gap in the literature on preferred DA architecture by interviewing survivors of HNC on the utility and potential visual designs for an eDA for patients with HNC to encourage informed and collaborative decision-making. Through phenomenological inquiry, the goal was to answer the following questions: (1) What do survivors of HNC think about the utility of electronic decision-support tools utilizing visuals for patients with HNC? and (2) What suggestions do survivors of HNC have on a potential design for visual features and core components of a prototype eDA?

Results will be used to inform next steps in the development and integration of eDAs in HNC care.

## Methods

### Prototype Visual Development

**Figure 1:**
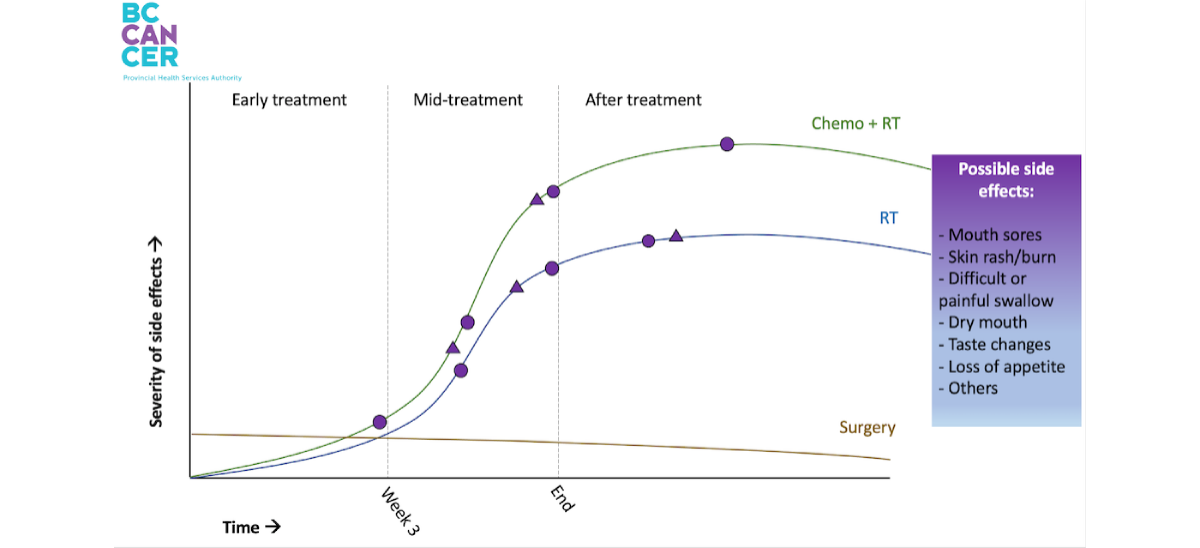
Severity of side effects graph for a prototype decision aid (displayed to patients during interviews). RT: radiation therapy.

**Figure 2:**
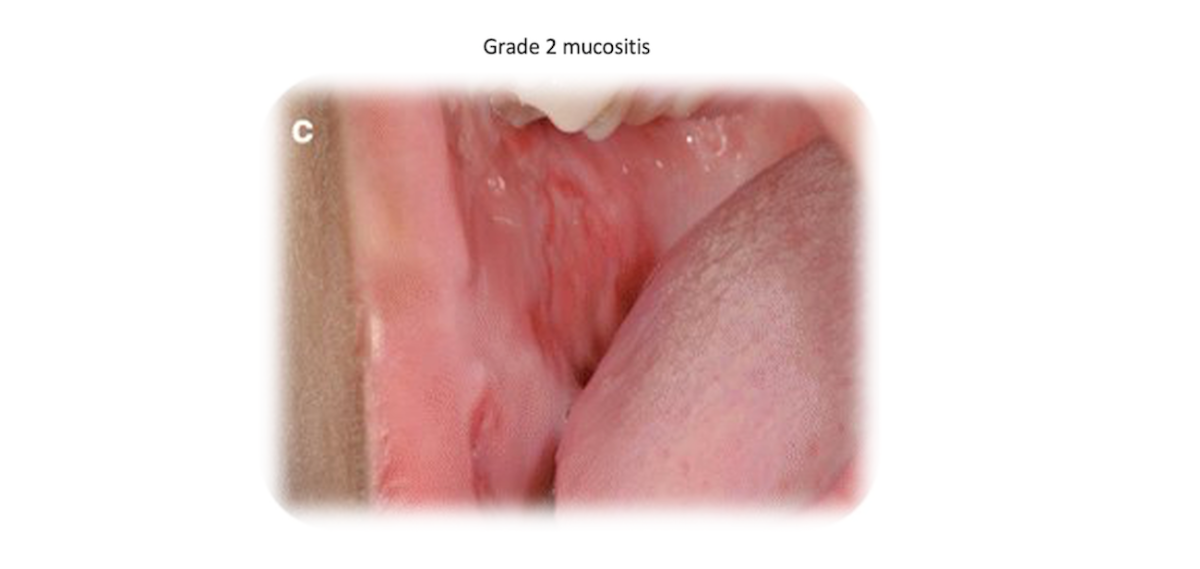
Example image with Common Terminology Criteria for Adverse Events grading and name (mucositis). Credit [10].

**Figure 3:**
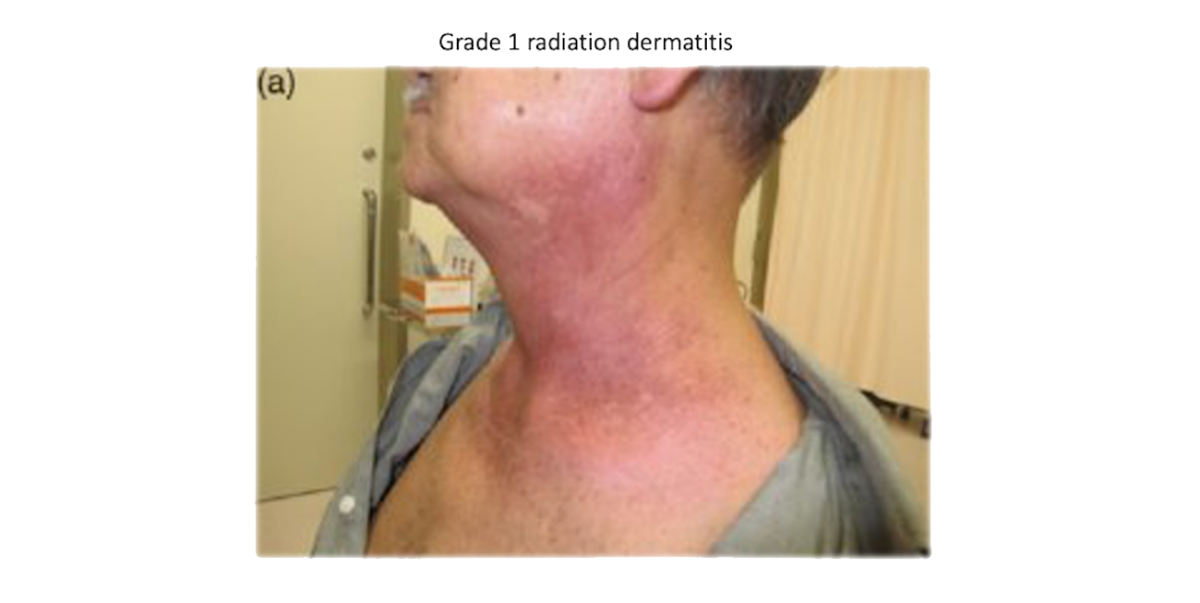
Example image with Common Terminology Criteria for Adverse Events grading and name (radiation dermatitis). Credit [11].

A point-of-reference (ie, example screen visualizations) mock-up of a DA was developed for the interviews (Figure 1), containing a graph used in the symptom management clinic at BC Cancer-Victoria (BCC-Vic). A line for surgery is included in the graph, serving as a placeholder only, as surgery is managed by surgeons external to BCC-Vic. The graph was displayed to participants in the interviews. Two symptoms were selected for visual demonstration, oral mucositis (Figure 2; photo credit [10]) and radiation dermatitis (Figure 3; photo credit [11]), noted by a shape on the curve. Hovering the cursor over these shapes triggers a pop-up image of the side effect, increasing in severity from left to right in accordance with the Common Terminology Criteria for Adverse Events (CTCAE) [12] grade. The information used in the design and graphs shown to participants was based on the evidence-based literature on HNC [13-15]. A key for these shapes was not provided, as they were meant to provide a demonstration of the mouse-over effect, where information could be obtained by moving the cursor over areas of the screen.

### Recruitment and Materials

Two phases of recruitment were conducted through convenience and purposive sampling, respectively, with a target of 6-12 participants or until no new knowledge was obtained [16]. Inclusion and exclusion criteria are described in Textbox 1. Upon consent, demographic, diagnosis, and treatment plans were extracted from BCC’s electronic medical record.

**Inclusion and exclusion criteria for the semistructured interview.**

**Inclusion criteria**

- Diagnosis of head and neck cancer (including nasopharyngeal) of any staging with completion of treatment (radiation or chemotherapy, with or without surgery)
- Treatment completed within the previous 5 years
- Resides on Vancouver Island or Gulf Islands

**Exclusion criteria**

- Inability to communicate proficiently in English
- Inability to meet via a virtual platform
- Participation discouraged by oncologist, psychiatrist, or other physician

### Interviews and Analysis

One-hour long, semistructured interviews, including field notes and reflective questions [17], were conducted following an interview guide. Interview questions were open ended and fell under the categories of introduction, background, experience, e-tools, evaluation, and conclusion. The interview questions were designed to elicit participants’ experience in learning about side effects of treatment along with their impression of the visuals presented to them (in terms of how well they helped explain treatment and side effects; see Multimedia Appendix 1 for the full interview script). For consistency, interviews were conducted by a single researcher (ES), who is most familiar with the subject area and experienced in patient interviews. No other researchers participated in the interviews to remove potential power dynamics that may disempower or affect participant opinions. Participants were introduced to the interviewer including their motivations and background behind this research.

Interviews were held virtually through Zoom (Zoom Video Communications, Inc.), to allow for screen sharing while also ensuring participants safety during COVID-19; all participants attended from their home. Participants were provided a demonstration of the eDA but did not interact with it. Participants were encouraged to ask for further demonstration if needed and were welcomed to use Zoom’s annotate feature. All interviews were video-recorded, transcribed, and analyzed using Taguette software [18,19]. Reflexive thematic analysis was performed on annotated transcripts, field notes, and reflections, where an open and iterative process was applied to coding following the 6-step approach of Braun et al [16]. A set of tagged codes (see Multimedia Appendix 2 for the codes) was used to identify concepts in the transcripts related to the research questions. These codes were used to develop concepts or “domain summaries at the start of the analytic process.” [16] Upon further engagement and critical reflection, related concepts were then grouped into themes [16]. Coding was completed by the researcher ES who was most familiar with the data and then verified by AWK. Data validation was conducted by sharing preliminary themes with participants for correction, modification, or confirmation.

### Ethics Review

Review Ethic Board (REB) approval was granted on January 18, 2021, from the University of Victoria Human REB (University of Victoria Study #BC20-0546) and UBC/BC Cancer Agency REB (H20-02307). All participants provided written informed consent to take part in the study.

## Results

### Demographics

A total of 12 participants were interviewed: 6 recruited from the Head and Neck Support Group at BCC-Vic and 6 identified by an oncologist. Participant ages ranged from 34 to 76 years, with half (n=6) of the participants diagnosed with stage III cancer and nearly half (5/12, 42%) undergoing surgery prior to radiation. As many as 7/12 (58%) participants were offered adjuvant chemotherapy: 3 accepted cisplatin and the remaining 4 declined chemotherapy; 9/12 (75%) participants accessed the symptom management team on a regular basis, while 10/12 (83%) connected with a counselor through treatment (Table 1).

**Table 1:** Participant demographics.

Characteristic		Frequency, n (%)
**Sex**		
	Male	8 (67)
	Female	4 (33)
**Age**		
	30-40	1 (8)
	41-50	2 (17)
	51-60	3 (25)
	61-70	2 (17)
	71-80	4 (33)
**Cancer staging^a^**		
	Stage I	2 (17)
	Stage II	4 (33)
	Stage III	6 (50)
	Stage IV	
**Treatment type**		
	Surgery	5 (42)
	Radiation	12 (100)
	Chemotherapy	3 (25)
	Cisplatin	3 (25)
	Cetuximab	0 (0)
	Declined by participant	4 (33)
**Degree of involvement with the symptom management team** **(general practitioner in oncology and registered nurse)**		
	Less than once per week	3 (25)
	Weekly	5 (42)
	Greater than once per week	4 (33)
**Degree of involvement with patient and family counseling**		
	None	2 (17)
	1-3 times through treatment	3 (25)
	Greater than 3 times through treatment	7 (58)

^a^Based on the American Joint Committee on Cancer 8th edition [20].

### Thematic Categories

A total of 61 codes were developed and applied, pointing to 17 concepts related to eDAs and the prototype. These 17 concepts were organized into 9 themes, separated into 2 categories (Table 2).

**Table 2:** Thematic categories and concepts.

Category	Themes	Concepts
eDAs^a^ and decision support	Familiarity with DAs^b^ Support of concept: usefulness and value of visual aids in explaining treatment and its side effects Versatility of prototype	Support of concept Appreciation for learning with visuals Communication Patients would use the app in different ways Design and features Emotion, traumatic experience, and resiliency Emotional impact of physical changes that can be seen by others Trend for a specific symptom to leave a large, lasting impact Coping strategies Altruism Meditation/mindfulness
Evaluation of mock-up	Reaction to protype Favorite features Preference for customizability Complexity Presentation type Suggestions for improvement	Recommendations Appreciation for the care team Areas for improvement within the care team List of practical suggestions Differences in experiences before and during COVID-19 Value of family and connecting with other patients

^a^eDA: electronic decision aid.

^b^DA: decision aid.

### Category 1: eDAs and Decision Support

The term “decision support tools” or “decision aids” was new to 75% (9/12) of participants, while 3/12 (25%) were familiar due to their line of work (Table 3). Regardless of eDA experience, there was strong support for the concept of using visual aids to enhance the explanation of treatment options, their potential side effects, and timeline, demonstrated by SUPPORT OF CONCEPT being the most frequently applied code (see Multimedia Appendix 2).

All participants expressed the usefulness and value of photographs, videos, and graphs. Learning styles appeared to influence the degree of value placed on graphics, with visual learners demonstrating the most enthusiasm. Those whose information needs were adequately met through their oncologist’s verbal explanation still suspected images would reinforce and amplify their message.

The codes USEFUL and VALUE were the most frequently tagged alongside PHOTOS and VIDEOS, often describing statements on how viewing photos of side effects would have clarified and adjusted treatment expectations. Lastly, several participants felt that viewing severe side effects prior to initiating treatment would serve as a catalyst, motivating prophylactic, intensive therapy and self-care strategies to optimize outcomes and quality of life. For example, one participant felt that viewing images of radiation dermatitis prior to treatment would have improved their compliance with skin care guidelines.

An unexpected theme was the potential versatility of eDAs in this area. Participants envisioned the eDA serving not only as an educational tool, but also as a communication tool to use both within and outside clinical encounters. Of the 12 participants, 3 (25%) felt that an eDA would prepare them for oncologist appointments by prompting the creation of informed questions. Participants also described their interest in using an eDA with the interdisciplinary care team, friends, and family. Two participants, for example, described the exhaustion that resulted from the draining task of updating loved ones on their day-to-day well-being.

**Table 3:** Representative quotes for thematic category 1: eDAs^a^ and decision support.

Theme and subthemes			Representative quotes		
1. Familiarity with DAs^b^			“That’s the whole point of my life is to provide the data for those tools.” “I have [heard of DAs] in terms of like breast cancer screening.”		
**2. Support of concept: usefulness and value of visual aids in explaining treatment and its side effects**			“I think an app would be awesome!” “And it seems like it’s really prime and the right time to be investing in more technologies, just because they’ve received such an expansion. Now, of course, everyone’s watching health dollars and how much healthcare is costing.” “It’s an exciting initiative, I think it’s great to try to incorporate technology into improving care and treatment.” “I like this, I like what you’re doing here. I like this concept very much. Good for you, you know, getting early into the treatment and looking at side effects. So this is very effective already.”		
	Visual learners	“I think it would be great for me because I’m a visual learner, but I think it’d be great for other people because, even those who are not visual learners, because I found it, like- I’m a reader, and I found it difficult to read at certain times and also things I read, I read it and then re-read it and I’d be like, ‘What does this even say?’ I couldn’t answer simple questionnaires, I was like, ‘I don’t even know what they’re asking’.” “I think visuals are very helpful, audio and visual you know if you have like a recording, or you have slides on your screen because I tend to be a visual learner. Anyway, and it’s something that you can actually re-read yourself. It’s very concrete. And it’s very well organized.” “I’m a visual learner so I, I really think that would benefit me, so yeah, very interesting.” “I think the use of pictures work for me. I’m a visual person so that would work for me, videos and depending on the topic I think videos are quite useful as well.”			
	Visual to augment the message	“[The prototype] would have augmented [the oncologist’s] message. So yeah, I think any visually would help.” “I remember they sent me in a room with one of the [radiation] technicians with a sheet of possible side effects. But it was very, again, it was very technical, and kind of, you know, ‘Do this. Don’t do that. You might have this. You can use this cream,’ but I think if there’s been a tool like this, for them to go through that would have been really helpful.” “If I can see pictures, or if I’m given handouts, or if the oncologist speaks more slowly, or shows me pictures. Yeah, that would have been more helpful to me.”			
	Clarifying and adjusting treatment expectations	“I think an image is worth a thousand words.” “I think seeing a picture like that would have been prepared me more.” “I like the graphic images that come up because then you can see ‘Oh yeah, these, these sores in my mouth or this burning in the neck area okay that’s what it looks like,’ and it’s like, ‘yeah, that’s what I went through.’” “And that really helps clarify things so, yeah, a lot of information.”			
**3. Versatility of a prototype**			“I’d want to study [the app] and then I’d have questions and then if I went in [to my appointment] and [my oncologist] said, ‘Oh, here’s what to expect,’ and I had already looked at it and could think of my questions.” “Early on, because you just don’t really understand what the process looks like. But if you have seen some of these photos, you might be like, oh okay I should ask questions about that.” “Yeah, definitely. And probably ahead I would want to look at it ahead of time, and then discuss.”		
	Interdisciplinary care team	“I would probably carry it with me to each medical appointment. Because you know, the first question is, ‘Well, how are you this week?’ So I’d just opening the chart and say, ‘Well I felt like I was here [pointing at chart] even though I should have been here [pointing]. Can you explain why I have not experienced this side effect?’ Yeah I think it’d been perfect.” “‘How are you feeling today?’ because everyone asks you those questions. And now you could just show them, ‘See I’m feeling this [pointing to chart]. This is how it’s changed on the [chart] today.’” “It would be helpful to bring it to the nurses, like, you know, during treatment I didn’t see my oncologist that often. The nurses would be helping me with most of these symptoms and things so they would have access to that as well maybe you could read it and discuss it with them.” “I personally liked it and I think it helps when you’re trying to pass [information] on to somebody else so we use that sort of system, again [in my work] for making decisions.” “There was a hard part of trying to explain symptoms that I had.”			
	Sharing with friends/family	“I think I would really want to share this [eDA] with the people close to me. And so that that would be pretty neat if rather than, you know, people say, ‘How are you doing?’ and you say, ‘Oh I’m doing good, I’m doing good,’ or ‘This is what’s happening,’ it would be neat to be able to share whatever the news is, with being able to show other people a graph of what’s happening.” “And then [my supports] would have a better idea. If you said, ‘I’m in week four.’ They might not know. Is this the end or just halfway through or...? But this provides some really good context to what you’re going through.” “My oncologist was just maybe a really good communicator, but like [using an eDA] didn’t seem necessary. But this would have been really helpful when I’m explaining things to other people, you know, when my parents were like, ‘What’s going to happen?’ Would be nice to have something like this to, to show them like it would help me explain it better.” “So I think the person who supports the people, because they’re going to look in your mouth and go, ‘That’s what’s happening.’ I think that’s normal right and maybe they even feel comforted that, that looks right you know.” “...encourage people to share it so that the people in their life like, get it because it’s hard to understand, you don’t see a lot of what goes on, it doesn’t show right it’s not like a cast.”			

^a^eDA: electronic decision aid.

^b^DA: decision aid.

### Category 2: Evaluation of Core Visual Components of a Prototype eDA

Initial reactions to the screens and visualizations that were presented to participants during the interviews were positive, with several participants exuding immediate enthusiasm. All participants described their favorite features in a desired prototype eDA. The visualizations presented were most praised for their simplistic design using a combination of pictures, colors, graphs, and text that offered a snapshot of treatment (Table 4).

Although there was unanimous agreement on the usefulness and value of visuals shown, the least consistent responses regarded the complexity of the graph. All participants felt they could read the graph without an explanation; some even read it “instantly.” While some felt the eDA visualizations may be too simplistic, one-third of participants (n=4) expressed a concern that the graph is overly complicated for the public, which includes individuals of varying education levels, ages, and health literacy. One participant expressed concern that the use of technology in medical care may be disconcerting to the elderly population, but suggested that if the difficulty is technology navigation, then the oncologist can operate the device.

Although there was strong support for the basic features of the prototype, every participant provided suggestions for improvement that pointed to their desire for customized information. Some mentioned features to consider customizing including treatment type (option to view a single treatment only), radiation dose (total radiation grays or number of fractions), filtering by side effect, adjustable timelines, results stratified by sex, detail within photos, option to display information source/reference, and associated statistics.

There were mixed opinions on the appropriateness of medically explicit images that become increasingly gruesome as the CTCAE grading score advances. Every participant agreed to view these images, but several appreciated the invitation to skip. Those in favor of including vivid detail argued that it is an accurate and honest display of treatment realities, while those hesitant to include graphic photographs were concerned that it would instill fear and possibly dissuade the pursuit of treatment. Two participants suggested offering the option of viewing an artistic sketch with less detail instead of a photograph. Another participant suggested emphasizing that symptoms are manageable with guidance from the health care team, using this as a segue to discuss supportive care measures such as medications and self-care techniques. Furthermore, they suggested it is “really important” to make clear that these strategies will “not cure it but will help them.”

There were mixed responses on which type of device (eg, desktop/laptop, tablet, phone) a prototype should be designed for, discussing the advantages and limitations of each device. Those with strong opinions based their response on how they envisioned themselves using an eDA. For example, one participant suggested a mobile phone interface for the benefit of portability, as they envisioned using it on public transport. A different participant preferred a computer to view details on a larger screen. Lastly, another participant thought a tablet to be the most practical for clinic use as it could be passed between the patient and health care provider while using a screen large enough for comfortable viewing.

SUGGESTIONS FOR IMPROVEMENT was the third most frequently applied code. Most recommendations fit into the following 7 categories:

- Additional information to add: Participants offered creative suggestions on information and features to add to the prototype, summarized in Table 5
- Legend: As previously described, 2 shapes dotted the graph representing 2 different side effects. These shapes were used for demonstration purposes only and would not be part of the final prototype, but participants found this confusing and were concerned that symbols would complicate the graph. They liked the idea of selecting the side effects of interest from a list on the side.
- Links to additional resources: Two participants highlighted the opportunity to link to supportive care resources, such as Inspire Health, BCC Patient and Family Counseling, and meditation exercises.
- List of side effects: Several participants recommended expanding the list of side effects to encompass rare side effects, such as fatigue, headaches, hair loss, neuropathy, tinnitus, vision problems, and dermatological changes.
- Interactive tracking: There was great enthusiasm for building out interactive tracking options. Some ideas were options to track diet intake, speech language pathologist–prescribed exercise routine, mental health, and symptoms such as pain and nausea.
- Add videos: Several participants suggested adding videos, with 1 participant particularly adamant on the value video could add, including “video clips of people who were able to speak to their experience.”
- Late side effect/life after treatment: Several participants requested additional information on life after treatment.

**Table 4:** Representative quotes for thematic category 2: evaluation of mock-up.

Theme and subthemes			Representative quotes		
4. Reaction to prototype			“I can tell you right off the bat, my reaction is this is fantastic. Oh, that’s really great! That was good.” “I like how clean and simple this is” “I like it. Great idea, and then that ended up just seeing that graph. Okay. It clicked. I, again, I think it’s awesome and again that’s just the way I do things the way I make decisions like that.” “Awesome! The graph to me, it’s wonderful.” “Gold star!”		
5. Favorite features			“This is brilliant, especially what you just described, if you could choose your side effects, or you could choose the side effects you’re concerned about like, you’re like, ‘I’m tired.’ Okay I got that I’m tired, that’s fine. I don’t need to learn more about that tired – I get that, but for the ones that are maybe, ‘Oh skin rash burn doesn’t sound good,’ maybe, you know, like the ones that seemed more serious or more concerning to be able to click on those because I was just thinking let’s say you had a shake for every possible side effects.” “Oh golly, well since you kind of explain the symbols and the curves and the free categories and time and all that. This is fantastic for my use because when you hover in the pictures come up then I can put it all together. And I think it’s very helpful because it’s important to know if you’re going to look in your mouth. And you see something that doesn’t look like your mouth used to look like. Then you can refer back to here and say, ‘Oh, I get it.’ Okay, did this happened to me during week for during week six during. Not at all. And yeah, I think this is very cool because it uses color is just a basic graph. It’s very simplistic but yet you know and then it hovers and you get photographs which you know every photograph almost looks like me. I could relate to all of that.” “I mean when I first looked at this without those the pop-up pictures, it’s good but then you just see that the severity goes up, but you can’t visualize it or you can’t internalize what that means. So I think being able to click on those pictures is great because that can give you an idea.” “[The prototype’s] essential because just looking at numbers, and like numbers in a row doesn’t really mean much. But looking at pictures, charts and graphs and however you want to display that information, particularly with color is a far better way of communicating content.” “You’re very smart to have been able to control when you show the photographs here, because you know a lot of presentations I have all the photographs and you just click on each photo and yet, I think that might frighten too many people away. Wo it’s good then that only when you hover is when the related photograph shows up.” “I like the chemo and radiation like to have them where they are parallel.” “I love the list of potential side effects”		
6. Complexity			“...dead simple, you know, the two axes the severity over time. Yeah, I don’t know, I’m biased by background and math and physics and things like that but you know that, you know, It makes it makes perfect sense” “This is pretty self-explanatory. You know, if I’m going through radiation, I’m just going to look at the blue line. It’s so simple.” “I think it’s good for adults, because it simplifies, and it just minimize this confusion when you break things down into words, pictures, colors, sound.” “Seriously, the KISS [Keep It Simple Stupid] theory. This is perfect, because the more fancy, you’re going to be bombarded with questions and complaints and who knows what. So I think the simpler the better.” “If you’re not well educated something like this could leave somebody awful confused.” “You have to assume there’s patients who have no education and they would not be able to make sense of this.” “I would worry about especially...about older patients with, you know, some might have cognitive or dementia. They would go, ‘What is this this? Some kind of mathematical thing? I don’t understand this.’ and they wouldn’t use it.” “It might be to assume the level of education, or sophistication of the patients. And if you want to make it accessible for everyone, then, then it might need a different might need to show it in a different way.” “I would just worry about the graph...especially for patients who have no scientific training whatsoever.” “It’s fine but, you know, not everybody relates to graphs.” “I like that idea of it but I would say, why would you need a graph if you’re going to have a visual? Why doesn’t one of you just have someone come on and say, ‘This is a picture that could happen you could look like this after three weeks, four weeks, you could look like this after five weeks,’ rather than a graph where someone is trying to figure out what the graph is.”		
**7. Preference for customizability**			“If there’s any ability to customize that would be a really useful feature.” “The simple graph is good, especially if there’s any way to customize it to be a little bit more like you check [the side effects you want to see].”		
	Support of graphic detail	“I like the graphic images that come up because then you can see, ‘Oh yeah, these sores in my mouth or this burning in the neck area, okay, that’s what it looks like’ and it’s like ‘yeah that’s what I went through.’”			
	Opposing detail	“[The oncologist] using this has to support that this is the worst-case scenario.” “I guess as long as a person knows these are possible side effects and not necessarily your degree of severity. Yeah, I guess that’s right. Yeah, no I think that’s good.” “I like the idea of that, but the only thing that you’ve given us is some fear factor a little bit. If you look in there and say, ‘Geez I don’t know if I should go through this because look what’s going on.’ Because I thought that’s what’s going to happen to me, but it didn’t. I was a bit sunburnt looking but nothing like what you showed there- nothing. And I didn’t get the most sores. So, as long as, ‘This is the worst case scenario.’” “I don’t mind those pictures. In fact, like I said when I look at that and go, ‘Well that didn’t happen to me. Wow-that’s bad! Like I’m glad I didn’t have that,’ but what if it didn’t happen here? What if yours was worse than that or, like, I think, pictures are really important, but maybe if someone had an option of either a drawing or a picture as opposed to a picture or no picture.” “So for people that are a little bit nervous or don’t know what the word is- they feel they get the upset stomach thing from seeing [graphic photos], if they had a picture, not a photo, I mean like an artist drawing, so that they have an idea...It doesn’t show the same thing- it’s not the same thing! But if someone can at least see a neck and then some dots on it, but they really don’t want to see someone’s neck burn because that’s going to make them sick, just to have that option...so everyone’s involved.”			
8. Presentation type			“I don’t use an iPad, and I do wonder how much is generational. I just use my phone and my laptop for work. But, like my parents, and I think that a lot of this cancer is in an older age group, that they use their iPads all the time. So I think for me it would be phone and laptop but I recognize that might be a generational thing. I don’t have an iPad. I think most people have phones, right, if they have any of those devices.” “For me personally, I have a preference to use it on the computer screen computer, like the regular desktop...I don’t like to carry a lot of electronics around with me, so I don’t always carry my phone.” “I’m 52, I’m going to choose a laptop anyway, because it’s bigger. I wear reading glasses. So I like to see it big.” “I’m looking at this on the phone right now and it makes perfect sense” “The bigger the screen, the more accessible.” “You can have something that’s able to be used in all three devices.”		
**9. Suggestions for improvement**			“Find ways to visually simplify it a little bit.” “Maybe you want to put a comments area” “I was thinking what’d be great on the app, if there could be a little pop up.”		
	Legend	“I just see those five [symbols on the prototype] and what the relationship they are. So, I really agree with not having too many because it was just a road of shapes you’d be like, you know, it’s too much, but to be able to choose it or to pick a few of the [side effects]” “I might do something different with that, like, I don’t know if triangles and circles are the best way”			
	Links to additional resources	“I was thinking it would be great on the app, if there could be a little pop up: ‘Don’t forget Inspire Health’ or whatever other resources, you would like to add to it.”			
	List of side effects	“I love the list of potential side effects but as I say I would include others if you know other like eyes, definitely.” “I love your suggestion of adding in the interactive tracking possibilities that would be very helpful”			
	Interactive tracking options	“I like tracking things and graphs. If it didn’t exist, I would do something on my own that made it interactive, so that I would track every day.”			
	Add videos	“I think video would be the way to go. It’s for a person my age. You read. What do you retain? Keep reading. I think if I could see somebody, the exercises they were performing. I think that would be much more informative and easier to grasp.” “I think it’s an excellent idea to have a video that people. The doctor, maybe can go say I’m just going to leave the room for a few minutes, watch this video for five minutes or whatever it is. And I think that’s a great idea and have the patient check that and, and, or have the doctor sitting there for doctrine to target wait to wait another five minutes, but I think is a great idea or I’ll send a video home with the patient.” “I’m a visual person so that would work for me, videos and depending on the, on the topic I think videos are quite useful as well.”			
	Late side effects/life after treatment	“I think that would be another thing is warning about late side effects.” “What I’m looking for is, you know, some sort of post treatment timeline that says at one year. Most of our people are here at two years, most of our people are here these are the some of the side effects they’re experiencing. Oh and look you know 40% of these people are having cognitive issues of some kind, just like they just get mentally tired more quickly than they used to do.”			

**Table 5:** Recommended additions to the prototype.

Recommendation	Representative quote
Include a clarifying disclaimer statement	“I would also want clarification that, that this is not, you’re not talking about prognosis here that this is side effects, because I think I might see a graph like that and be initially afraid”
Add photos of the radiation bed, chemo room, and other commonly used clinical areas	“...have a visual a picture of what the treatment room looks like”
Extend the timeline	“The only thing I would add is maybe like a line vertical bar for vertical line for one year out another one for 10 years ago.” “My thinking is that those dotted gray lines could move like I don’t mean you should move them I mean they could”
Integrate self-care and coping strategies	“I do think that some kind of an app [could be linked]. Even that could allow you to practice some techniques for coping with the actual treatment, like I said, like a breathing where you could watch a short video on.”
Add an area for feedback	“I was gonna say maybe you want to put a comments area but none that’s another good idea because adults always have comments, but that’s about it.”
Add an option to view statistics	“I’m interested in things like well, of all your patients what percent just did RT, what percentage did chemo and RT what you know of all those combinations surgery chemo. How did it break out, like, you know, what are the what are the treatment paths that people have been on. What are the result pass for those treatment combinations and what are the side effects down the road. I’m a data person so I wanna, I want to make sure there’s the data behind that”
Advertise other relevant research and clinical trials	“I like your suggestion to have seen if there’s a way to link it with that other study that’s currently going on where you’re inputting things.”

## Discussion

### Principal Findings

This study contributes to the literature on the utility and patient-recommended requirements and design of eDAs. Participant feedback was overwhelmingly supportive of using visuals to support explanations of treatment and potential side effects. All participants could imagine themselves using an eDA as an education and communication tool and agreed to contribute to the next phase in the full development of eDA, further demonstrating their endorsement.

### Use and Value of Visual Aids

Literature demonstrates that patients prefer images to demonstrate benefit and harm trade-offs in health as they are perceived as easier to understand [21]. Verbally relayed numerical facts were least effective in encouraging a specific health decision while graphical information was the most preferred, demonstrating that “consideration should be given to developing visual aids to support shared clinical decision making” [21]. This is consistent with our findings: every participant agreed that the use of images alongside verbal explanations would complement or augment an oncologist’s message. Furthermore, “using transparent information formats enhances risk comprehension, communication, and recall and helps people make better decisions about their health” [22].

### Malleability of the eDA

Participants were interested in adding interactive options; most were intrigued by symptom tracking to auto-populate a personalized curve on the graph. Here lies an opportunity to incorporate patient-reported outcome measures, standardized instruments used to capture patients’ perceptions of their health status, functional status, or health-related quality of life [23]. Integrating patient-reported outcome measures within the eDA would support the gold standard of patient self-administration without interviewer interpretation [24]. Furthermore, literature demonstrates the benefit of real-time symptom tracking during cancer care, improving health outcomes and communication with health care providers [25,26].

When considering participants’ desired features alongside cumulative feedback, customizability is required to suit the range of learning styles, needs, and preferences. A potential solution to varying information needs may include suggestions from Table 5 and then allowing users to filter and sort the viewable data. For example, participants unanimously supported the idea of selecting side effects of interest from an extensive list of potential side effects. Taken further, graphics could be shown as real-life photos or sketched images to better meet participants comfort level with medically explicit images. Embedding customizability may also address the discourse regarding the suitability of the graph. The mixed responses align with the literature on graph literacy, which “reveal[s] that people, regardless of their numeracy skills, differ substantially in their ability to understand graphically presented quantitative information about health” [22]. Furthermore, Nayak et al [27] tested graphical interpretation of a visual dashboard on patients with prostate cancer and despite 78% of participants having a college education, variation remained in graph literacy results.

### Prognosis Versus Quality of Life

Our visualizations designed and presented to participants did not include information on prognosis or survival outcomes to simplify the inceptive eDA. The information presented was designed to be used in the presence of an oncologist who could personalize the message and discuss prognosis on a case-by-case basis. Previous research, however, demonstrated that comprehension of medical information on survival and cancer treatment options is equal or better than when patients are shown the same information with the addition of mortality statistics [28]. In a viewpoint paper on his personal experience with advanced stage tongue cancer, Kushniruk [29] argues for “patients to be more informed about choices and statistics, including the meaning of survival curves in relation to different treatment options.” Although the desire for incorporation of information about prognosis in DAs was not a prominent theme in our findings, the importance of survival cannot be overlooked and should be considered for inclusion in future iterations.

### Limitations

Selection bias was introduced through the convenient sampling method. Recruitment initially targeted patients at BCC-Vic’s Monthly Head and Neck Support Group, comprising those closely matching the inclusion criteria. This group, however, may more likely represent survivors who experienced challenges through treatment, thus introducing a source of bias. To help offset this, an equal number of participants (n=6) were recruited through purposive sampling to diversify the demographics. Because of the nonprobability sampling methods, the results cannot be generalized to a wider population. Future work should consider including the perspective of those without cancer (ie, cancer naïve) and survivors along the cancer trajectory (ie, never diagnosed through to long-term survivorship). Furthermore, race was not included in the demographics and future work should include diverse racial representation, including historically underrepresented groups.

There are limitations to the mock-up design method used for presenting design ideas and visualizations that did not adhere to a formal development process, such as the Ottawa Patient Decision Aid Development eTraining or the International Patient Decision Aid Standards Collaborations [30]. It was instead based on the research teams’ phenomenological and personal experiences with HNC; graphs used with patients at BCC-Vic; informal, preliminary feedback from patients; and results of the scoping review. The designs and visualizations were sufficient for this study purpose of applying a user-centered design [31] to this innovation with plans to build through the co-design methodologies of Kushniruk and Nøhr [32] and Kushniruk and Patel [33].

Repeat interviews to improve data richness were not conducted as these interviews were intended to be preliminary, setting the stage for the next phase of the project that will include in-depth workshops with participants. Additionally, rich data were collected from the single interviews and additional interviews were not yet deemed necessary.

Lastly, due to the specificity of the target population, results cannot be generalized to other tumor types. Further requirements analysis and testing will be required prior to expanding to other populations.

### Conclusions

This research highlights the value of incorporating visuals into technology-based innovations to support patient decision-making in oncology care. All participants felt an eDA, used with their oncologist, would support an enhanced and transparent explanation of treatment and augment the quality of the consult. Participants liked the simple design of the prototype visualization but desired customizability to adapt the eDA to their individual information needs. This research highlights the value of user-centered design, rooted in acceptability and utility, in medical health informatics, recognizing cancer survivors as the ultimate knowledge holders.

The next steps include applying the co-design methodologies of Nayak et al [27] and Kushniruk [29] to develop a fully functioning eDA to be tested with patients with cancer, incorporating visualizations and the feedback described in this paper. This will consider implications to clinical workflow, including physician’s time, and improve accessibility by ensuring visualization is amendable to printing for those without digital access. Usability testing of the eDA will be conducted in a clinical setting at BCC-Vic and the University of Victoria through a newly funded follow-up study (ie, the Head and Neck Cancer Application for Patients and their Partners [HANC APP Study]).

## Appendix

Multimedia Appendix 1 Interview questions.

Multimedia Appendix 2 Frequency of tagged codes.

## Abbreviations

BCC-Vic: BC Cancer-Victoria
CTCAE: Common Terminology Criteria for Adverse Events
DA: decision aid
eDA: electronic decision aid
HNC: head and neck cancer
REB: Review Ethic Board

## References

[1] Patterson RH, Fischman VG, Wasserman I, Siu J, Shrime MG, Fagan JJ, Koch W, Alkire BC (2020). Global Burden of Head and Neck Cancer: Economic Consequences, Health, and the Role of Surgery. Otolaryngol Head Neck Surg.

[2] (2013). Head and Neck Cancers. National Cancer Institute.

[3] Brown R, Butow P, Wilson-Genderson M, Bernhard J, Ribi K, Juraskova I (2012). Meeting the decision-making preferences of patients with breast cancer in oncology consultations: impact on decision-related outcomes. J Clin Oncol.

[4] Drew P, Chatwin J, Collins S (2001). Conversation analysis: a method for research into interactions between patients and health-care professionals. Health Expect.

[5] Fang CY, Heckman CJ (2016). Informational and Support Needs of Patients with Head and Neck Cancer: Current Status and Emerging Issues. Cancers Head Neck.

[6] Garcia-Retamero R, Galesic M, Garcia-Retamero R, Galesic M (2013). Chapter 14 Guidelines for Transparent Communication in a Globalized World. Transparent Communication of Health Risks: Overcoming Cultural Differences.

[7] Stacey D, Légaré France, Lewis K, Barry MJ, Bennett CL, Eden KB, Holmes-Rovner M, Llewellyn-Thomas H, Lyddiatt A, Thomson R, Trevena L (2017). Decision aids for people facing health treatment or screening decisions. Cochrane Database Syst Rev.

[8] Stringer E, Kushniruk AW (2021). Utility of electronic decision-support tools for patients with head and neck cancer: A scoping review. Knowledge Management & E-Learning: An International Journal.

[9] Petersen JF, Berlanga A, Stuiver MM, Hamming-Vrieze Olga, Hoebers F, Lambin P, van den Brekel Michiel W M (2019). Improving decision making in larynx cancer by developing a decision aid: A mixed methods approach. Laryngoscope.

[10] Vitale Marina Consuelo, Modaffari Carola, Decembrino Nunzia, Zhou Feng Xiao, Zecca Marco, Defabianis Patrizia (2017). Preliminary study in a new protocol for the treatment of oral mucositis in pediatric patients undergoing hematopoietic stem cell transplantation (HSCT) and chemotherapy (CT). Lasers Med Sci.

[11] Zenda Sadamoto, Ishi Shinobu, Akimoto Tetsuo, Arahira Satoko, Motegi Atsushi, Tahara Makoto, Hayashi Ryuichi, Asanuma Chie (2015). DeCoP, a Dermatitis Control Program using a moderately absorbent surgical pad for head and neck cancer patients receiving radiotherapy: a retrospective analysis. Jpn J Clin Oncol.

[12] US Department of Health and Human Services (2017). Common Terminology Criteria for Adverse Events (CTCAE) Version 5.0. National Cancer Institute.

[13] Hunter M, Kellett J, Toohey K, D'Cunha NM, Isbel S, Naumovski N (2020). Toxicities Caused by Head and Neck Cancer Treatments and Their Influence on the Development of Malnutrition: Review of the Literature. Eur J Investig Health Psychol Educ.

[14] Memorial Sloan Kettering Cancer Center (2023). Radiation Therapy to the Head and Neck. Memorial Sloan Kettering Cancer Center.

[15] Galloway T, Amdur RJ (2023). Management and prevention of complications during initial treatment of head and neck cancer. UpToDate.

[16] Braun V, Clarke V, Hayfield N, Terry G, Liamputtong P (2019). Chapter 48 Thematic Analysis. Handbook of Research Methods in Health Social Sciences.

[17] Nathan S, Newman C, Lancaster K, Liamputtong P (2019). Qualitative Interviewing. Handbook of Research Methods in Health Social Sciences.

[18] Taguette, the free and opensource qualitative data analysis tool. Taguette.

[19] Rampin V, Rampin R (2021). Taguette: open-source qualitative data analysis. Journal of Open Source Software.

[20] Amin MB, Greene FL, Edge SB, Compton CC, Gershenwald JE, Brookland RK, Meyer L, Gress DM, Byrd DR, Winchester DP (2017). The Eighth Edition AJCC Cancer Staging Manual: Continuing to build a bridge from a population-based to a more "personalized" approach to cancer staging. CA Cancer J Clin.

[21] Goodyear-Smith F, Arroll B, Chan L, Jackson R, Wells S, Kenealy T (2008). Patients prefer pictures to numbers to express cardiovascular benefit from treatment. Ann Fam Med.

[22] Galesic M, Garcia-Retamero R (2011). Graph literacy: a cross-cultural comparison. Med Decis Making.

[23] U.S. Department of Health and Human Services FDA Center for Drug Evaluation and Research, U.S. Department of Health and Human Services FDA Center for Biologics Evaluation and Research, U.S. Department of Health and Human Services FDA Center for Devices and Radiological Health (2006). Guidance for industry: patient-reported outcome measures: use in medical product development to support labeling claims: draft guidance. Health Qual Life Outcomes.

[24] Appleby J, Devlin N, Parkin D (2016). Using Patient Reported Outcomes to Improve Health Care.

[25] Hong YA, Hossain MM, Chou WS (2020). Digital interventions to facilitate patient-provider communication in cancer care: A systematic review. Psychooncology.

[26] Patel RA, Klasnja P, Hartzler A, Unruh KT, Pratt W (2012). Probing the benefits of real-time tracking during cancer care. AMIA Annu Symp Proc.

[27] Nayak JG, Hartzler AL, Macleod LC, Izard JP, Dalkin BM, Gore JL (2016). Relevance of graph literacy in the development of patient-centered communication tools. Patient Educ Couns.

[28] Zikmund-Fisher BJ, Fagerlin A, Ubel PA (2010). A demonstration of ''less can be more'' in risk graphics. Med Decis Making.

[29] Kushniruk A (2019). The Importance of Health Information on the Internet: How It Saved My Life and How it Can Save Yours. J Med Internet Res.

[30] O'Connor A, Stacey D, Saarimaki A (2023). Ottawa Patient Decision Aid Development eTraining. The Ottawa Hospital.

[31] Dopp AR, Parisi KE, Munson SA, Lyon AR (2019). A glossary of user-centered design strategies for implementation experts. Transl Behav Med.

[32] Kushniruk A, Nøhr Christian (2016). Participatory Design, User Involvement and Health IT Evaluation. Stud Health Technol Inform.

[33] Kushniruk AW, Patel VL (2004). Cognitive and usability engineering methods for the evaluation of clinical information systems. J Biomed Inform.

