# Supplementary material for: Decision Aids for Patients With Head and Neck Cancer: Qualitative Elicitation of Design Recommendations From Patient End Users
Source: JMIR Hum Factors. 2023 Jun 5;10:e43551. doi: 10.2196/43551 (PMC10280338; doi:10.2196/43551)
Supplement: Multimedia Appendix 1 [file humanfactors_v10i1e43551_app1.docx]

| **Topic** | **Time allotment** | **Questions/ statements** |
| --- | --- | --- |
| Introduction | 5 mins | Welcome, introduction to interviewer, overview of study purpose, reminder of confidentiality and right to pass on any questions |
| Background | 5 mins | Did you request copies of any of your medical records?  Did you conduct self-research? |
| Experience | 10 mins | What do you recall being told about treatment side effects and was your experience with side effects as you anticipated? |
| E-tools | 10 mins | Overview of what they are, purpose  What do you think about the use of visuals (pictures and videos) in helping explain treatment and its potential short- and long-term side effects?  Do you think visual images such a pictures or videos would have had a different impact than the verbal explanation you received from your oncologist?  Would a better understanding of side effects have influenced your feelings towards treatment?  In retrospect, would you do anything differently? |
| Evaluation | 15 mins | Present mock-up design  What are your thoughts on this as a potential design?  Anything lacking or excessive?  How would you like it shown? (tablet, etc)  User friendly?  Can you imagine yourself using it when discussing treatment with your oncologist?  What would you change? |
| Conclusion | 5 mins | Any final thoughts or comments?  Consent for follow up interview?  Thank you |
