# Supplementary material for: Decision Aids for Patients With Head and Neck Cancer: Qualitative Elicitation of Design Recommendations From Patient End Users
Source: JMIR Hum Factors. 2023 Jun 5;10:e43551. doi: 10.2196/43551 (PMC10280338; doi:10.2196/43551)
Supplement: Multimedia Appendix 2 [file humanfactors_v10i1e43551_app2.docx]

| **Code** | **N of tags** | **Frequency** |
| --- | --- | --- |
| Support of concept | 129 | 7.2% |
| Information need | 105 | 5.9% |
| Prototype suggestions | 93 | 5.2% |
| Side effect | 90 | 5.0% |
| Useful | 79 | 4.4% |
| Told about tx | 72 | 4.0% |
| Life after tx | 63 | 3.5% |
| Gratitude | 61 | 3.4% |
| Value | 58 | 3.2% |
| Coping | 55 | 3.1% |
| Emotion | 54 | 3.0% |
| RO | 51 | 2.8% |
| Photos | 49 | 2.7% |
| Preparation | 49 | 2.7% |
| Decision | 48 | 2.7% |
| Quote | 44 | 2.5% |
| Self research | 42 | 2.3% |
| Communication | 41 | 2.3% |
| Video | 37 | 2.1% |
| Giving back | 35 | 2.0% |
| Shock | 34 | 1.9% |
| Appearance | 33 | 1.8% |
| Family | 29 | 1.6% |
| Pt ideas | 28 | 1.6% |
| Optimism | 26 | 1.5% |
| Devices | 25 | 1.4% |
| EHR | 25 | 1.4% |
| Fear | 25 | 1.4% |
| Traumatic | 25 | 1.4% |
| Pt to pt | 22 | 1.2% |
| Positive Outcome | 19 | 1.1% |
| RT | 19 | 1.1% |
| Mindfulness/meditation | 17 | 0.9% |
| RD | 16 | 0.9% |
| RN | 16 | 0.9% |
| Surgeon | 15 | 0.8% |
| Not wanting to know | 12 | 0.7% |
| Confusing | 11 | 0.6% |
| Disappointment | 11 | 0.6% |
| MO | 10 | 0.6% |
| Panic | 10 | 0.6% |
| Shows pic | 10 | 0.6% |
| Chemo | 9 | 0.5% |
| Experience w/ DAs | 9 | 0.5% |
| Graphics | 8 | 0.4% |
| Sad | 8 | 0.4% |
| Enthusiasm | 7 | 0.4% |
| PFC | 7 | 0.4% |
| Skeptism | 7 | 0.4% |
| Dentist | 6 | 0.3% |
| Privacy | 6 | 0.3% |
| Inspire Health | 5 | 0.3% |
| SLP | 5 | 0.3% |
| Vulnerability | 5 | 0.3% |
| COVID | 4 | 0.2% |
| Depression | 4 | 0.2% |
| Resiliency | 4 | 0.2% |
| GPO | 2 | 0.1% |
| QOL | 2 | 0.1% |
| Locum | 1 | 0.1% |
| Timing | 1 | 0.1% |
